# HSA21 Single-Minded 2 (Sim2) Binding Sites Co-Localize with Super-Enhancers and Pioneer Transcription Factors in Pluripotent Mouse ES Cells

**DOI:** 10.1371/journal.pone.0126475

**Published:** 2015-05-08

**Authors:** Audrey Letourneau, Gilda Cobellis, Alexandre Fort, Federico Santoni, Marco Garieri, Emilie Falconnet, Pascale Ribaux, Anne Vannier, Michel Guipponi, Piero Carninci, Christelle Borel, Stylianos E. Antonarakis

**Affiliations:** 1 Department of Genetic Medicine and Development, University of Geneva Medical School, Geneva, Switzerland; 2 Department of Biophysics, Biochemistry and General Pathology, Seconda Università di Napoli, Napoli, Italy; 3 Division of Genomic Technologies, RIKEN Center for Life Science Technologies, Yokohama, Japan; 4 University Hospitals of Geneva, Geneva, Switzerland; 5 iGE3 Institute of Genetics and Genomics of Geneva, Geneva, Switzerland; Michigan State University, UNITED STATES

## Abstract

The HSA21 encoded Single-minded 2 (SIM2) transcription factor has key neurological functions and is a good candidate to be involved in the cognitive impairment of Down syndrome. We aimed to explore the functional capacity of SIM2 by mapping its DNA binding sites in mouse embryonic stem cells. ChIP-sequencing revealed 1229 high-confidence SIM2-binding sites. Analysis of the SIM2 target genes confirmed the importance of SIM2 in developmental and neuronal processes and indicated that SIM2 may be a master transcription regulator. Indeed, SIM2 DNA binding sites share sequence specificity and overlapping domains of occupancy with master transcription factors such as SOX2, OCT4 (Pou5f1), NANOG or KLF4. The association between SIM2 and these pioneer factors is supported by co-immunoprecipitation of SIM2 with SOX2, OCT4, NANOG or KLF4. Furthermore, the binding of SIM2 marks a particular sub-category of enhancers known as super-enhancers. These regions are characterized by typical DNA modifications and Mediator co-occupancy (MED1 and MED12). Altogether, we provide evidence that SIM2 binds a specific set of enhancer elements thus explaining how SIM2 can regulate its gene network in neuronal features.

## Introduction

Down syndrome (DS) results from trisomy of human chromosome 21 (T21). It is the most frequent live-born aneuploidy, affecting 1 in 750 newborns. DS patients are characterized by a broad range of phenotypes including mental retardation, short stature, muscle hypotonia, congenital heart defects or Alzheimer disease neuropathology [1]. Among the HSA21 genes, transcription factors are important candidates to explain some DS features. Indeed, transcription factors are known to play a global role in the gene transcription regulation via their direct or indirect binding to promoter and enhancer elements. Consequently, their dysregulation (in trisomic cells for instance) is likely to impact the expression of the target genes leading to the perturbation of a variety of distinct molecular pathways. More than 20 transcription factors or transcription regulators map on HSA21 and may directly or indirectly contribute to the transcriptional regulation [2]. Among them, Single-minded 2 (SIM2) appears to be a relevant candidate to explain some DS features, in particular the cognitive impairment.

SIM2 is a member of the basic helix-loop-helix Per-Arnt-Sim (bHLH/PAS) family of transcription factors. The proteins of this family contain a basic DNA binding domain adjacent to a helix-loop-helix region and a PAS region, essential for the dimerization of the proteins and the proper formation of active transcription factor complexes [3]. They are known to be involved in multiple fundamental biological processes including neurogenesis, hypoxic response, circadian rhythms or toxin metabolism [3–5]. The first single-minded protein was identified in *Drosophila melanogaster* as a key regulator of the midline cell development in the central nervous system (CNS) [6–8]. Interestingly, the Drosophila Sim does not only contribute to gene activation in the midline cells [7] but also to indirect gene repression in the lateral CNS, through activation of repressive factors [9, 10]. To form active complexes, the Drosophila Sim protein dimerizes with another member of the bHLH-PAS family called Tango [11]. The SIM proteins identified in mammals show a high degree of similarities with their Drosophila homolog [12–16]. They contain comparable bHLH and PAS domains and dimerize with the Tango ortholog called ARNT (Ah receptor nuclear translocator). The presence of ARNT is essential for the formation of active complexes since SIM2 does not homodimerize [3]. The murine *Sim2* is expressed early during development in many tissues affected in DS such as developing forebrain, ribs, vertebrae, limb skeletal muscles or kidney [17]. Similarly, the human *SIM2* is expressed during the early fetal life in the central nervous system and in key brain structures involved in learning and memory processes [15, 18]. The expression pattern of *SIM2* and its known function in Drosophila suggest that it may be a good candidate to explain some of the DS cognitive features. Interestingly, the transgenic mice harboring three copies of *Sim2* exhibit some of the DS phenotypes namely a moderate impairment of learning and memory as well as a reduced exploratory behavior and sensitivity to pain [19–21]. *Sim2* -/- mutant mice die rapidly after birth due to breathing failure and display rib, vertebral and craniofacial abnormalities [22, 23].

In order to better understand how SIM2 can participate in some DS features, we have further explored its regulatory role in mammalian cells. An accurate list of SIM2 target genes in normal and trisomic cells is required for understanding its role in genetic regulation. We have mapped the SIM2 DNA binding sites using chromatin immunoprecipitation followed by high-throughput sequencing (ChIP-Seq) in a mouse embryonic stem cell (mES cell) line that stably overexpresses *Sim2* under the control of an inducible system. Using this model, we have identified 1229 regions occupied by SIM2 and showed that the associated target genes fulfill molecular functions related to the DS phenotypes. More importantly, we observed that a significant fraction of SIM2 binding sites overlaps with genomic regions occupied by master transcription factors involved in the genetic control of the pluripotent state, namely SOX2, OCT4, NANOG or KLF4. These regions are characterized by typical enhancer signatures and our data demonstrate that the binding of SIM2 could also predict a super-enhancer activity. Altogether, we provide new evidence of the SIM2 functional capacity.

## Materials and Methods

### Cell culture

The EBRTcH3 (EB3) parental mouse ES line and the clones overexpressing Sim2-Flag have been previously described in [24, 25].

mES cells were grown on 0.1% gelatin (Sigma #G1890) coated dishes in DMEM high glucose medium (Life technologies #41965) supplemented with 15% Fetal Bovine Serum (FBS HyClone, Thermo Scientific #SH30070), 2mM L-glutamine (Life technologies #25030), 1mM Sodium pyruvate (Life technologies #11360), 100units/ml penicillin/streptomycin (Bioconcept #4-01F00-H), 0.1mM 2-mercaptoethanol (Life technologies #31350), 1000units/ml Leukemia Inhibitory Factor (LIF, Millipore #ESG1107) and 1μg/ml tetracycline (Sigma #T7660). Cells were incubated at 37°C in a 5% CO_2_ atmosphere. Medium was changed every day and cells were passed every 1 or 2 days using 1X Trypsin-EDTA (Sigma #T4174).

### Induction of *Sim2* transgene expression

Culture medium was changed for tetracycline-free medium three hours before passing the cells in order to eliminate the residual tetracycline. Cells were passed using 1X Trypsin-EDTA (Sigma #T4174). Five million cells were plated in each new dish and cultured in the tetracycline-free medium for 26h starting from passage time.

### Fluorescence Activated Cell Sorting

Cells were grown in presence or absence of tetracycline for 26 hours and collected using Trypsin-EDTA. Pellets were washed with PBS and 300’000 cells from each line were collected in 300μl of PBS supplemented with 2% FBS for the measure of Venus fluorescence by FACS (FACSCalibur platform).

### RNA preparation

Total RNA was isolated 26h post induction, concurrently with the crosslinking experiment. RNA samples were prepared using TRIzol reagent (Life technologies #15596) as per the manufacturer’s instructions. Quality was assessed using the Agilent 2100 Bioanalyzer (RNA 6000 Nano Kit, #5067) and quantity was measured on a Qubit instrument (Life technologies). RNA was extracted from each of the SIM2 clones (A6, B8 and C4) and from three independent cultures of the EB3 clone.

### Reverse-Transcription PCR

Reverse transcription was performed using 1μg of total RNA and the SuperScript II Reverse Transcriptase (Life technologies #18064). PCR was performed on 1μl of cDNA diluted 10 times using the following primers: TTCGAATGAAGTGCGTCTTG (forward) and ACATGTTGCTGTGGAGCTTG (reverse) for mSim2 and TGCCTCATCTGGTACTGCTG (forward) and GAACATGCTGCTCACTGGAA (reverse) for mArnt. The PCR program was the following: 94°C for 5min followed by 10 cycles of 94°C for 30s, 60°C (∆-1) for 30s, 72°C for 30s, followed by 25 cycles of 94°C for 30s, 50°C for 30s, 72°C for 30s and a final elongation step at 72°C for 7min.

### Chromatin immunoprecipitation (ChIP)

ChIP was performed using the SimpleChIP Enzymatic Chromatin IP Kit (Cell Signaling #9003) according to manufacturer’s instructions. Briefly, mES cells SIM2 (A6, B8 and C4) and mES cells EB3 were cultured in absence of tetracycline for 26h. After the induction, 50 million cells from each clone were crosslinked using 1% formaldehyde. Digestion of the chromatin was performed with 5μl of Micrococcal Nuclease for 20 minutes at 37°C, followed by sonication (3 sets of 10-second pulses at 10% amplitude on a Branson Digital Sonifier 450). Independent chromatin immunoprecipitations were performed on SIM2 (A6, B8 and C4) and EB3 chromatin preparations using the equivalent of 20μg of chromatin DNA per IP. Each chromatin preparation was incubated at 4°C overnight with 6μg of anti-FLAG M2 antibody (Sigma #F3165). The next day, samples were incubated with 30μl of Protein G Magnetic Beads for 2h at 4°C, beads were washed and chromatin was eluted from the antibody/Protein G beads complexes. A 2% input sample of each chromatin preparation was saved before the immunoprecipitation as a control. Both input and eluted chromatin samples were reverse crosslinked in presence of Proteinase K for 2 hours at 65°C and DNAs were purified on columns.

ChIP experiments against OCT4, SOX2, NANOG, MED1 and MED12 were performed in the same conditions (same cross-linked pellets) on SIM2 A6 cells and EB3 cells using 6μg of the following antibodies: anti-Nanog (D2A3) XP (Cell Signaling #8822), anti-Oct3/4 (N-19) (Santa Cruz #sc-8628), anti-Sox2 (Santa Cruz #sc-17320), anti-Med1 (CRSP1/TRAP220) (Bethyl #A300-793A) and anti-Med12 (Bethyl #A300-774A).

### ChIP-Sequencing

Preparation of the libraries for high-throughput sequencing was performed using the ChIP-Seq DNA sample Prep Kit (Illumina #IP-102-1001), following the manufacturer’s instructions with some modifications. Libraries were prepared starting from 1.08ng of DNA from SIM2 (A6, B8 and C4) anti-FLAG ChIP, SIM2 (A6, B8 and C4) input, EB3 anti-FLAG ChIP and EB3 input samples. 8ng of starting material were used from ChIP and input DNA from OCT4, SOX2, NANOG, MED1 and MED12 ChIP experiments (Sim2 A6 and EB3 cells). Enrichment of the DNA fragments by PCR was performed using reagents and adapters from the TruSeq RNA Sample Preparation kit (Illumina #RS-122-2001) according to the following program: 98°C for 30s followed by 18 cycles of 98°C for 10s, 60°C for 30s and 72°C for 30s, followed by a final elongation step at 72°C for 5min. PCR clean up was done on Agencourt AMPure XP beads (Beckman Coulter #A63880). Libraries were validated on an Agilent Technologies 2100 Bioanalyzer (DNA1000 chip). Libraries were sequenced on Illumina HiSeq 2000, in single-end sequencing 1x36bp or 1x50bp (4 samples per lane).

### mRNA-Sequencing

mRNA-Sequencing libraries were prepared from 500ng of total RNA using the TruSeq RNA Sample Preparation kit (Illumina #RS-122-2001) following Illumina’s instructions. Libraries were sequenced on one lane of the Illumina HiSeq 2000 in paired-end sequencing 2x100bp.

### Co-immunoprecipitation experiments

SIM2 A6 cells and EB3 cells were grown in tetracycline-free media for 26h and harvested using Trypsin-EDTA. Total protein extract was collected in lysis buffer (50mM Hepes pH 8, 200mM NaCl, 0.1mM EDTA pH 8, 0.5% NP-40, 10% glycerol and protease inhibitors) after 1h at 4°C and centrifugation for 30min at 4°C (13000rpm). 50μl of beads (Dynabeads protein G, Life Technologies #10003D) were prepared for the immunoprecipitation by coupling with 2μg of antibody for 30min at room temperature. Immunoprecipitation was performed overnight at 4°C using 500μg of protein extract and the beads coupled to the following antibodies: anti-Sox2 (Y-17) (Santa Cruz #sc-17320), anti-Oct3/4 (N-19) (Santa Cruz #sc-8628), anti-Klf4 (R&D systems #AF3158), anti-Nanog (N-term and C-term) (Bethyl #A310-110A). Beads were washed four times for 5min at 4°C in 50mM TrisHCl pH 8, 250mM NaCl, 1% Triton X-100. Elution was performed in 200mM TrisHCl pH 8, 6% SDS, 15% glycerol, 3% ß-mercaptoethanol for 10min at 95°C. 5μl of the immunoprecipitated extract were analyzed by western blot using an anti-Flag antibody coupled to HRP (Sigma #A8592, 1:1000 dilution). Each experiment was performed in duplicate.

### ChIP-Sequencing analysis

For each sequenced library, reads generated from the sequencing were mapped against the mouse genome (mm9) using the BWA (Burrows-Wheeler Aligner) [26] alignment program with the default parameters (allowing 2 mismatches). Mapped reads were submitted to the HOMER (Hypergeometric Optimization of Motif EnRichment) software (http://biowhat.ucsd.edu/homer/ngs/index.html) [27] for the identification of SIM2 DNA binding sites. HOMER was used with the default parameters after removing of the duplicated reads. For each SIM2 clone, peak finding was done first by comparing the SIM2 tags to the input tags (background removal) and second by deleting all the non-specific sites identified in the EB3 control experiment. The genome ontology and motif discovery analyses were performed using HOMER.

Each identified peak was assigned to the closest gene(s) by calculating the distance separating the center of the peak from the TSS. All peaks located in intergenic or intronic regions were assigned to both the closest upstream and downstream genes. Peaks located in exonic or promoter regions were assigned to the unique gene to which they belong.

For OCT4, SOX2, NANOG, MED1 and MED12, peak finding was performed in HOMER by comparing the ChIP tags to the input tags in SIM2 A6 cells and EB3 cells independently. Each peak was then assigned to the closest gene by calculating the distance separating the center of the peak from the TSS.

### mRNA-Sequencing analysis

mRNA-Seq reads were mapped against the mouse genome (mm9) using the default parameters of BWA. For each gene, a custom pipeline was used to calculate the exon coverage. This coverage was normalized in reads per kilobase per million (RPKM). Differential expression analysis between *Sim2* expressing cells (A6, B8, C4) and EB3 control cells (3 independent replicates) was performed using the default parameters of EdgeR [28]. A gene was considered differentially expressed if the false discovery rate (FDR) was below 5%.

### Gene Ontology and Gene Set Enrichment Analysis (GSEA)

Gene ontology analyses were performed using DAVID (Database for Annotation, Visualization and Integrated Discovery) [29, 30]. Gene Set Enrichment Analysis was performed using the GSEA software [31]. Genes were sorted according to their fold change between *Sim2* expressing cells and EB3 cells (mRNA-Seq data). The GSEA analysis consisted in testing if a particular gene set was randomly distributed in this ranked list or enriched at the beginning or the end of the distribution. A positive enrichment score (ES) reflects enrichment in the upregulated genes whereas a negative ES reflects enrichment in the downregulated genes. This enrichment was considered significant if the FDR corrected p-value was less than 0.05 (after 1000 or 10000 permutations).

### ChIA-PET analysis

ChIA-PET data were taken from Zhang *et al*. [32]. Circular representation of the inter-chromosomal and intra-chromosomal interactions was done using the RCircos package (https://bitbucket.org/henryhzhang/rcircos).

### Overlap between Sim2 binding sites and other features

Enrichment around Sim2 DNA binding sites was performed using the ChIP-Cor Analysis Module of the ChIP-Seq Web Server (http://ccg.vital-it.ch/chipseq/documents.php). The relative abundance of each tested feature is reported in a 40kb window around the SIM2 DNA binding sites by comparing the position of the SIM2 peaks with the position of the target features. SOX2, NANOG, OCT4, MED1 and MED12 peak positions were taken from our ChIP-seq data in *Sim2* expressing cells. Other transcription factor peak coordinates were taken from Chen *et al*. [33] (after lift over of the data to mm9). Control MED1 peak coordinates were taken from Kagey *et al*. [34]. Chromatin modification marks were taken from the mouse ENCODE data in the UCSC genome browser mm9 build (http://genome.ucsc.edu/): P300 and PolII data are from ES-Bruce4 cells (LICR), H3K4me1 and H3K27ac data are from ES-E14 cells (LICR), H3K4me3 data are from ES-E14 cells (SYDH) and DNAseI HS data are from ES-E14 cells [17].

Overlap between each feature and the SIM2 DNA binding sites was tested using the windowBed command of bedtools, by using a 100bp window interval. The significance of the association was tested using a Fisher’s exact test comparing the number of features overlapping with the SIM2 binding sites and the number of features overlapping with a random set of 1229 intervals. The F-score was calculated according to [35].

### Accession number

Sequencing data have been submitted to GEO under the accession number: GSE59379.

## Results

### Identification of SIM2 DNA binding sites

**Fig 1:**
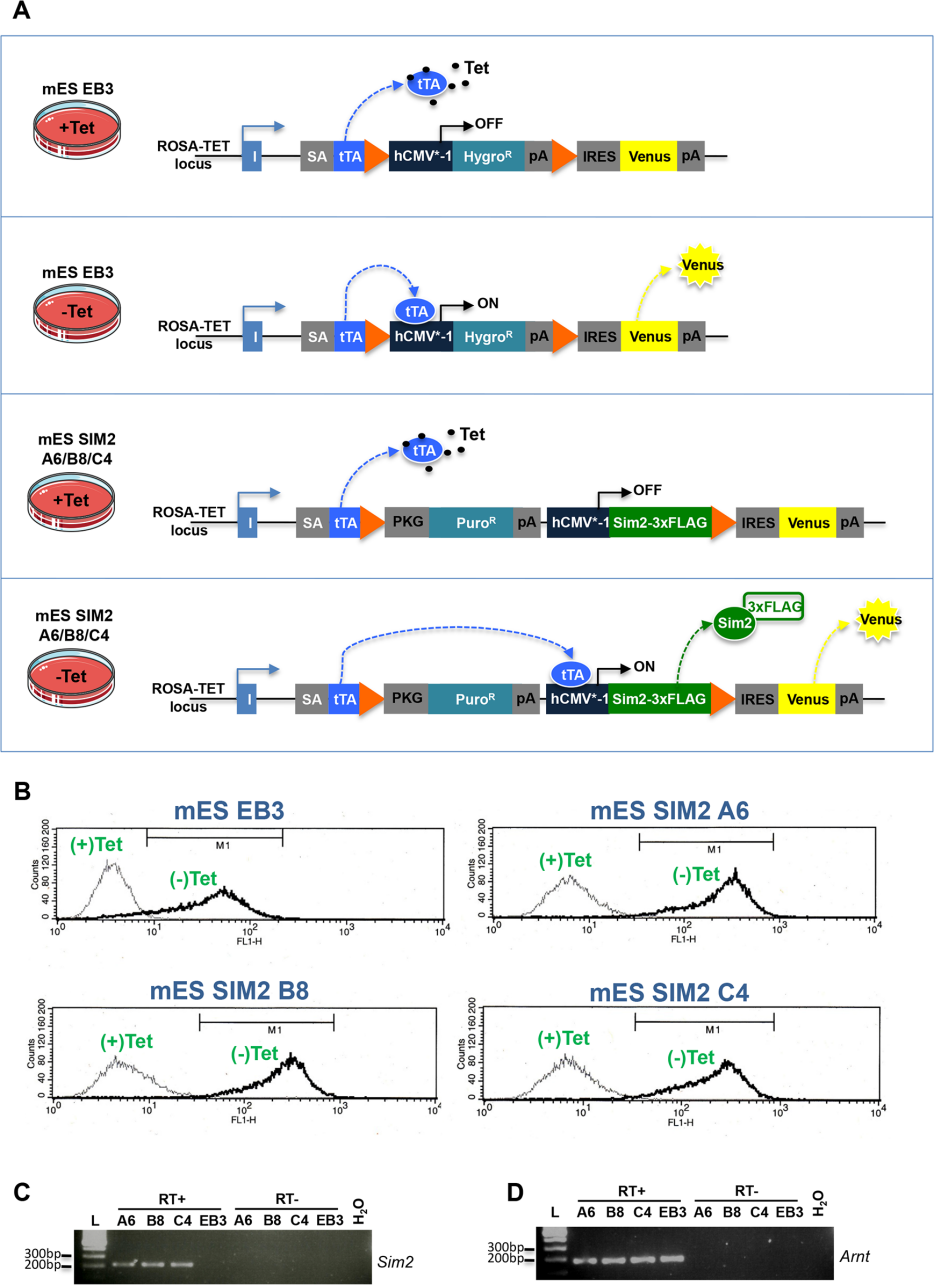
Cellular model. **a.** Schematic representation of the inducible ROSA-TET system. The mES SIM2 clones A6, B8 and C4 contain a Flag-tagged version of the mouse *Sim2* gene under the control of a modified human CMV promoter (hCMV*-1). In presence of tetracycline in the culture media (+Tet), the tetracycline-regulatable transactivator (tTA) is trapped and cannot bind the hCMV*-1 promoter. Upon removal of tetracycline (-Tet), the tTA binds the hCMV*-1 promoter inducing the expression of the *Sim2*-Flag-IRES-Venus construct. The mES EB3 parental line does not contain the *Sim2*-Flag transgene. The puromycin-resistant (Puro^R^) and hygromycin-resistant (Hygro^R^) cassettes are used for the clone selection process. SA: Splice Acceptor; pA: poly-adenylation site; IRES: Internal Ribosome Entry Site; orange triangles represent loxP sites. Modified from [25] **b.** Fluorescence-activated cell sorting (FACS) analysis of *Sim2* expressing and non-expressing clones. Cells were grown in the presence (+Tet) or absence (-Tet) of Tetracycline during 26 hours. The y-axis represents the number of cells and the x-axis the fluorescence intensity. **c and d.** Agarose gel electrophoresis results of reverse-transcription PCR assay (RT). Total RNA from +Tet cells was reverse transcribed and amplified using primers specific to *Sim2* (c) or *Arnt* (d) in presence (RT+) or absence (RT-) of reverse transcriptase. L: loading marker; H_2_O: PCR negative control.

We used of a mES cell line that stably overexpresses a Flag-tagged version of the mouse *Sim2* gene [24]. This model is based on a ROSA-TET system allowing the inducible overexpression of the *Sim2*-FLAG transgene upon removal of tetracycline from the culture media. We analyzed three different mES clones harboring the *Sim2* construct (named A6, B8 and C4) as well as the EBRTcH3 (EB3) parental line as a negative control (Fig 1A). The *Venus* transgene, inserted downstream of the construct, was used in the four lines as an internal control to verify the inducible system. A FACS (Fluorescence Activated Cell Sorting) analysis revealed that 26 hours of induction were sufficient to promote the expression of *Venus* in the four lines (Fig 1B). We confirmed the presence of *Sim2* transcripts in the induced A6, B8 and C4 expressing clones as opposed to the EB3 parental line by Reverse-Transcription PCR (RT-PCR, Fig 1C). Finally, we showed that the *Arnt* partner, essential for the formation of active transcription factor complexes, was expressed in all the lines (Fig 1D).

**Fig 2:**
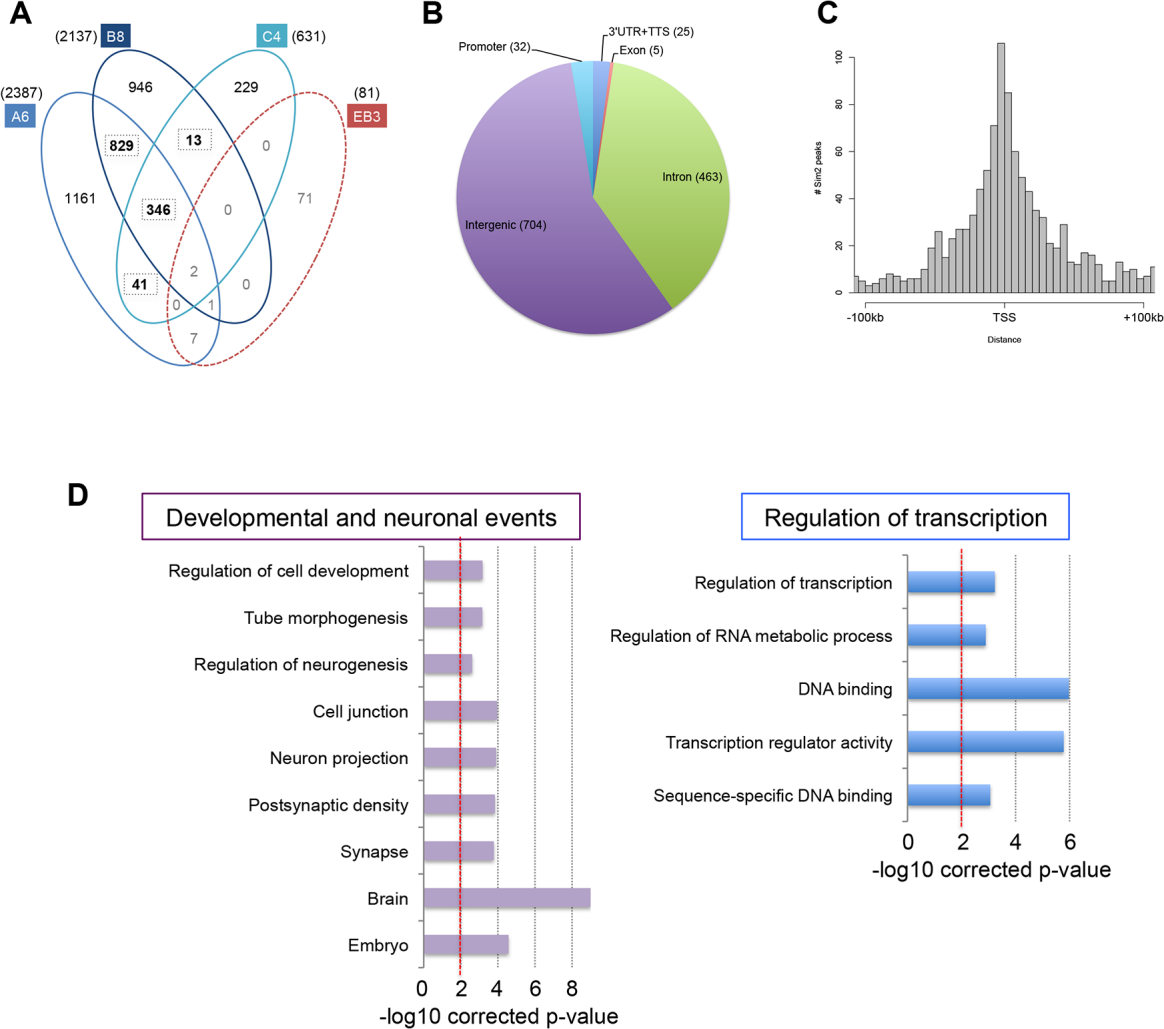
Identification and characterization of the SIM2 DNA binding sites by ChIP-seq. **a.** Venn diagram of the number of SIM2 binding sites identified by ChIP-seq in each SIM2 clones (A6, B8, C4) and EB3 line. The sum of the bold numbers is equal to the 1229 SIM2 DNA binding sites found in at least 2 SIM2 clones. **b.** The pie chart shows the genomic distribution of these 1229 sites. **c.** Distribution of the distances between the SIM2 DNA binding sites and the closest transcription start site (TSS). **d.** Selection of gene ontology terms significantly over-represented in the list of genes associated to a SIM2 DNA binding site.

We then performed ChIP-Sequencing in the A6, B8, C4 and EB3 lines after 26 hours of induction in tetracycline-free medium by using an anti-Flag antibody. ChIP and input DNAs were sequenced on the Illumina HiSeq 2000. 36 to 68 million reads were generated and mapped against the mouse genome (mm9) using the BWA aligner (Burrows-Wheeler Aligner) [26]. We then used HOMER [27] to analyze the reads and identify the SIM2 binding sites. We used the A6, B8 and C4 clones as biological replicates and identified 2387, 2137 and 631 peaks in each line, respectively (Fig 2A). After exclusion of the non-specific peaks detected in the EB3 parental line, we selected the binding sites that were identified in at least 2 replicate experiments. We described a total of 1229 SIM2 specific binding sites, including 346 sites common to the 3 *Sim2* expressing lines (S1 Table). The majority of these 1229 peaks were located in intergenic (57%) and intronic (37%) regions of the genome (Fig 2B). We found that 80% of the SIM2 peaks were located in a 100kb window around a known transcription start site (TSS) (Fig 2C). A total of 32 SIM2 binding sites were found in promoter regions defined by a window of -1kb/+300bp around the TSS (Fig 2B).

### Characterization of the putative SIM2 target genes

In order to identify putative SIM2 target genes, we assigned each of the 1229 peaks to the closest gene(s) by calculating the distance separating the center of the peak from the TSS. Peaks located in intergenic or intronic regions were assigned to both the closest upstream and downstream TSSs whereas peaks located in exons or promoter regions were assigned to a unique gene. In total, 1992 different genes were associated to one or more SIM2 binding site(s). We used DAVID (Database for Annotation, Visualization and Integrated Discovery) [29, 30] to perform a gene ontology analysis and check if this list of genes was enriched for specific biological functions (Fig 2D and S2 Table). Interestingly, the analysis revealed a significant enrichment for genes involved in developmental processes and more specifically in neurogenesis, including regulation of cell development (Benjamini corrected p-value p = 6.69e-04), tube morphogenesis (p = 6.89e-04), regulation of neurogenesis (p = 0.002) or regulation of nervous system development (p = 0.002). The same analysis revealed the over-representation of genes expressed in brain and embryonic tissues (p = 9.36e-10 and p = 2.66e-05, respectively) as well as cellular components such as synapse (p = 1.65e-04) or neuron projection (p = 1.24e-04) (S2 Table). These results confirmed the role of SIM2 in developmental events, specifically in the nervous system. Additionally, we found that those SIM2-associated genes were also significantly involved in mechanisms of transcription regulation, as revealed by gene ontology terms such as regulation of transcription (p = 5.78e-04), DNA binding (p = 1.06e-06) or transcription factor activity (p = 5.81e-06). These results show that SIM2 can control the expression of other transcription factors in the genome, suggesting that it may be an important master regulator. Interestingly, the list of SIM2 targets was also enriched for genes involved in cancer pathways, as revealed by the KEGG pathway analysis (p = 1.46e-04) (S2 Table). This finding is consistent with the reported involvement of SIM2 in several cancers [36–40].

### Validation by mRNA-sequencing and ChIA-PET data analyses

**Fig 3:**
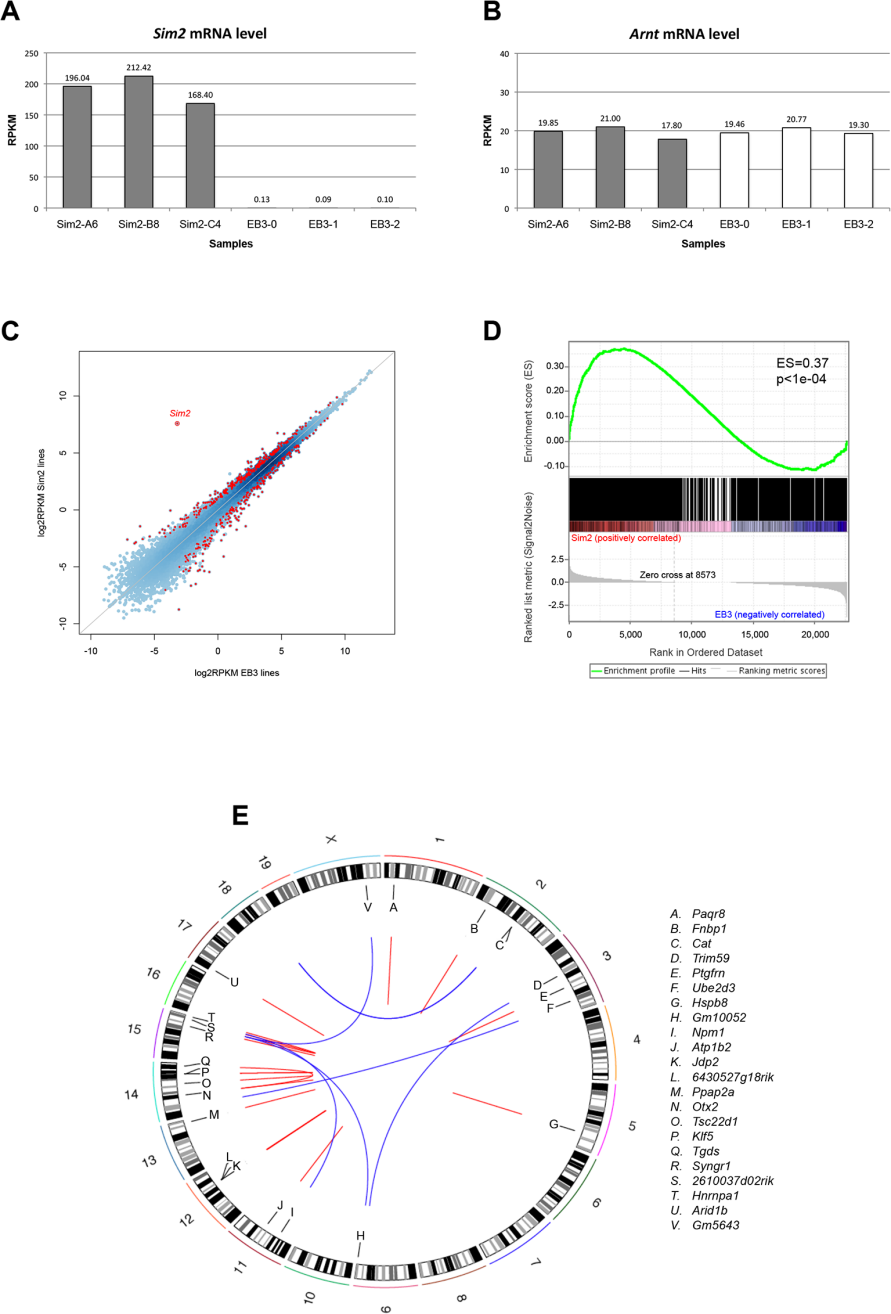
mRNA-sequencing and ChIA-PET analyses. *Sim2* (**a**) and *Arnt* (**b**) mRNA levels (RPKM) in A6, B8, C4 SIM2 clones and three EB3 replicates. **c.** Comparison of the gene expression level (mean log2 RPKM between the 3 replicates) between *Sim2* expressing cells (y-axis) and EB3 control cells (x-axis). Each blue dot represents a gene; differentially expressed genes (EdgeR FDR<0.05) are shown in red. The diagonal line represents the expected distribution of genes equally expressed between *Sim2* expressing and non-expressing cells. **d.** Enrichment of Sim2 targets among the genes upregulated in *Sim2* expressing clones as revealed by the GSEA analysis. Genes were sorted according to their expression fold change between *Sim2* expressing and non-expressing cells (x-axis, 0 showing the most upregulated gene). Black vertical bars show the position of the SIM2 targets in the ranked list. The enrichment score (ES in green) significantly deviates from zero at the beginning of the distribution showing that the SIM2 targets are not randomly distributed in the ranked list but enriched among the most upregulated genes. p: FDR corrected p-value **e.** ChIA-PET interactions occurring between SIM2 DNA binding sites and promoters of genes differentially expressed in *Sim2* expressing cells compared to EB3 cells (FDR<0.05). Blue lines show inter-chromosomal interactions and red lines intra-chromosomal interactions.

In order to further validate the SIM2 target genes, we have investigated the changes of mRNA levels induced by the overexpression of *Sim2*. We used mRNA-sequencing to study the transcriptome of the A6, B8, C4 and EB3 lines. Total RNA was collected concurrently with the ChIP-Seq experiment and sequenced on the Illumina instrument. The reads generated were mapped against the mouse genome using BWA and normalized in RPKM (Reads per Kilobase per Million) in order to compare the expression level of each gene between *Sim2* expressing and non-expressing cells. We first verified the expression level of *Sim2* in both conditions and confirmed its overexpression in A6, B8 and C4 (196.04, 212.42 and 168.40 RPKM, respectively) as opposed to the EB3 cells which showed very low levels of endogeneous *Sim2* transcripts (0.13, 0.09 and 0.10 RPKM in each of the 3 replicates, respectively) (Fig 3A). We also confirmed that *Arnt*, the SIM2 co-factor, is expressed at similar levels in all cell lines, with an average RPKM level of 19.7 (Fig 3B). This shows that the overexpression of SIM2-FLAG does not influence the level of endogenous *Arnt*. The SIM2-FLAG activity is therefore limited by the endogenous levels of *Arnt*, restricting the formation of active complexes to physiological ranges.

We then used EdgeR [28] to perform a differential expression analysis between *Sim2* expressing and non-expressing cells. This analysis revealed that 300 RefSeq genes were significantly upregulated and 230 genes significantly downregulated when *Sim2* is overexpressed in mES cells (FDR<0.05) (Fig 3C and S3 Table). However, the gene ontology analysis did not show enrichment for a specific type of biological functions (data not shown). A Gene Set Enrichment Analysis (GSEA) revealed that the SIM2 targets were significantly enriched among the most upregulated genes in *Sim2* overexpressing cells (Enrichment Score ES = 0.37, FDR<1e-04, Fig 3D), with 18.7% of the differentially expressed genes associated to a SIM2 DNA binding site. Interestingly, 99 genes previously associated to a SIM2 peak were found significantly dysregulated (24 down- and 75 up-regulated) in *Sim2* expressing cells (FDR<0.05) and can be considered as direct targets of SIM2 (S4 Table). A gene ontology analysis revealed that this list of 99 genes was not enriched for any particular biological functions (data not shown).

Importantly, SIM2 may also bind genomic regions that are not located in a direct proximity of a target gene. Indeed, the formation of chromatin loops within the nucleus is known to promote the interaction between promoters and regulatory regions located distantly to regulate the gene transcription. The ChIA-PET method (Chromatin Interaction Analysis by Paired-End Tag sequencing) has been developed to identify such long-range interactions [41, 42]. We used datasets of ChIA-PET chromatin interactions associated with RNA polymerase II available in mouse ES cells [32] to investigate the existing interactions between the SIM2 binding loci and distant gene promoters. Among the 1229 genomic regions bound by SIM2, 206 were found to physically interact with one or several gene promoter(s) occupied by a RNAPII transcriptionally active complex in mES cells (S5 Table). We observed 102 inter-chromosomal interactions, suggesting that SIM2 could act in *trans* to regulate the expression of distant targets. In contrast, 265 interactions occur between loci located on the same chromosome. Most of these intra-chromosomal interactions (63%) connected SIM2 binding loci and gene promoters distant from less than 100kb. Overall, the RNA polymerase II ChIA-PET datasets identified 310 unique transcribed gene promoters that physically interact with at least one SIM2 binding site. Interestingly, 22 of those genes were significantly dysregulated by the overexpression of *Sim2* (EdgeR FDR<0.05) (Table 1). We considered those as putative SIM2 targets since their expression level is associated with the binding of SIM2 in their promoter region (Fig 3E).

**Table 1:** List of Sim2 target genes identified using the ChIA-PET data.

Sim2 target			Associated Sim2 peak	Distance from peak (bp)
Gene name	logFC	FDR		
*2610037D02RIK*	-1.38	4.26E-02	Merged-chr15-96084454-1	29,585
*6430527G18RIK*	0.68	1.33E-02	Merged-chr12-88165302-1	60,462
*Arid1b*	0.65	7.12E-03	Merged-chr17-4948580-1	46,493
*Atp1b2*	0.90	2.51E-03	Merged-chr11-69395541-1	23,903
*Cat*	0.55	2.68E-02	Merged-chr18-65738596-1	Inter (chr2-chr18)
			Merged-chr18-65741928-1	Inter (chr2-chr18)
*Fnbp1*	0.70	1.70E-02	Merged-chr2-30916463-1	81,065
*Gm10052*	-0.64	7.38E-03	Merged-chr15-88698126-1	Inter (chr9-chr15)
*Gm5643*	-0.57	2.00E-02	Merged-chr15-88698126-1	Inter (chrX-chr15)
*Hnrnpa1*	-0.52	4.26E-02	Merged-chr15-88698126-1	14,372,818
*Hspb8*	0.76	2.59E-02	Merged-chr5-116858161-1	14,712
*Jdp2*	1.21	1.45E-02	Merged-chr12-86962262-1	21,897
			Merged-chr12-86961177-1	20,812
*Klf5*	0.81	2.23E-04	Merged-chr14-99739553-1	41,644
			Merged-chr14-99739095-1	41,186
*Npm1*	-0.48	4.07E-02	Merged-chr15-97424436-1	Inter (chr11-chr15)
*Otx2*	-1.90	5.44E-12	Merged-chr14-49274868-1	12,320
*Paqr8*	1.10	1.31E-02	Merged-chr1-20857728-1	22,969
*Ppap2a*	0.63	2.62E-02	Merged-chr13-113561150-1	29,951
*Ptgfrn*	0.62	1.67E-02	Merged-chr3-100967534-1	53,333
*Syngr1*	0.86	8.95E-04	Merged-chr15-80049889-1	128,116
*Tgds*	-0.82	3.31E-02	Merged-chr14-49274868-1	69,257,119
*Trim59*	-0.51	4.26E-02	Merged-chr9-110849737-1	Inter (chr3-chr9)
*Tsc22d1*	0.77	9.81E-05	Merged-chr14-76915540-1	100,773
*Ube2d3*	-0.78	9.81E-05	Merged-chr14-20569154-1	Inter (chr3-chr14)

### SIM2 co-localizes and interacts with master transcription factors

**Fig 4:**
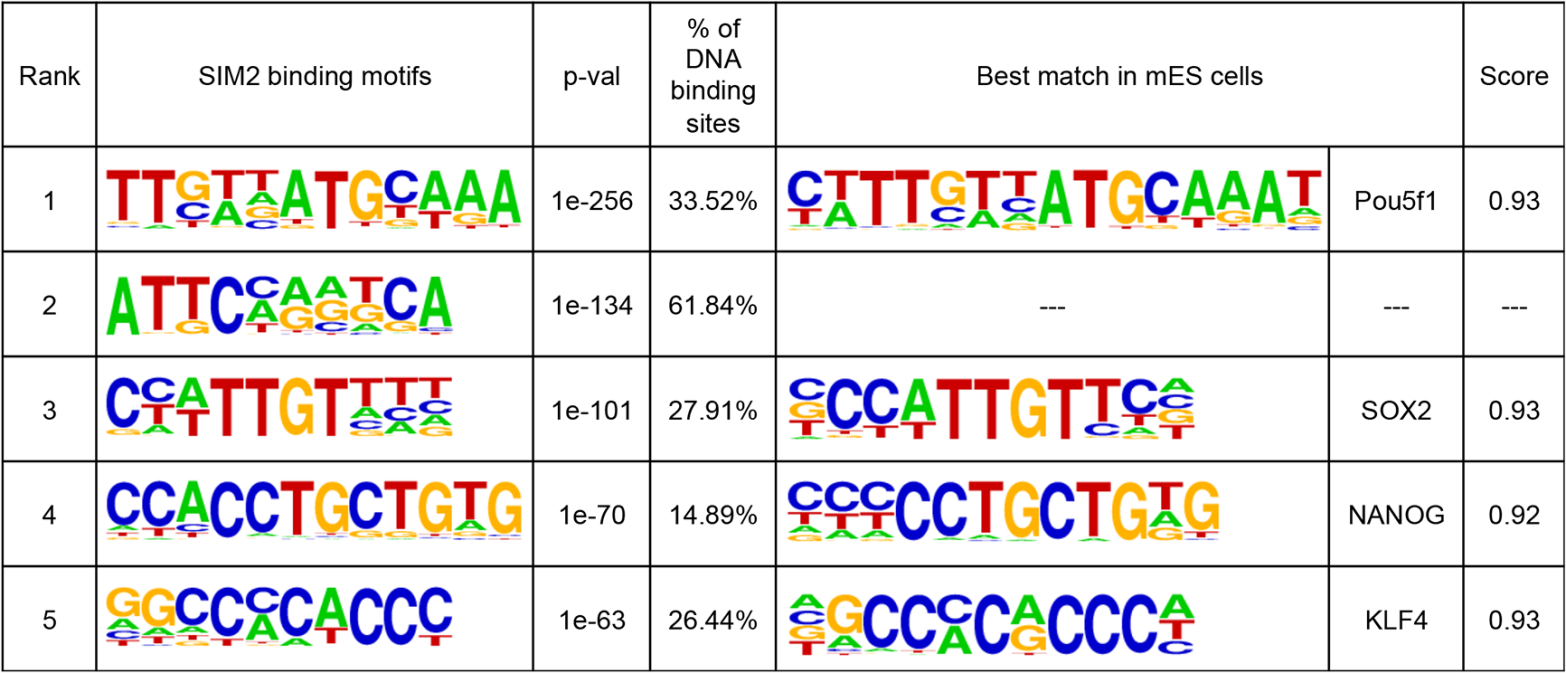
Motifs enriched in Sim2 DNA binding sites.

We then investigated whether SIM2 preferentially binds to specific DNA motifs. Using the HOMER algorithm, we identified five motifs significantly enriched in the SIM2 DNA binding sites (p-value<1E-50, Fig 4). Similar motifs were found when we independently analyzed peaks located in promoters, gene bodies or intergenic regions (data not shown). Interestingly, four of those enriched motifs were highly similar to motifs previously described in mouse ES cells for the binding of master transcription factors involved in the control of pluripotency: OCT4, SOX2, NANOG and KLF4 (Fig 4). Three of them (OCT4, SOX2 and NANOG commonly called OSN) are known to constitute the core of all mechanisms regulating the transcription program of ES cells and participating in the maintenance of their pluripotent state [43].

**Fig 5:**
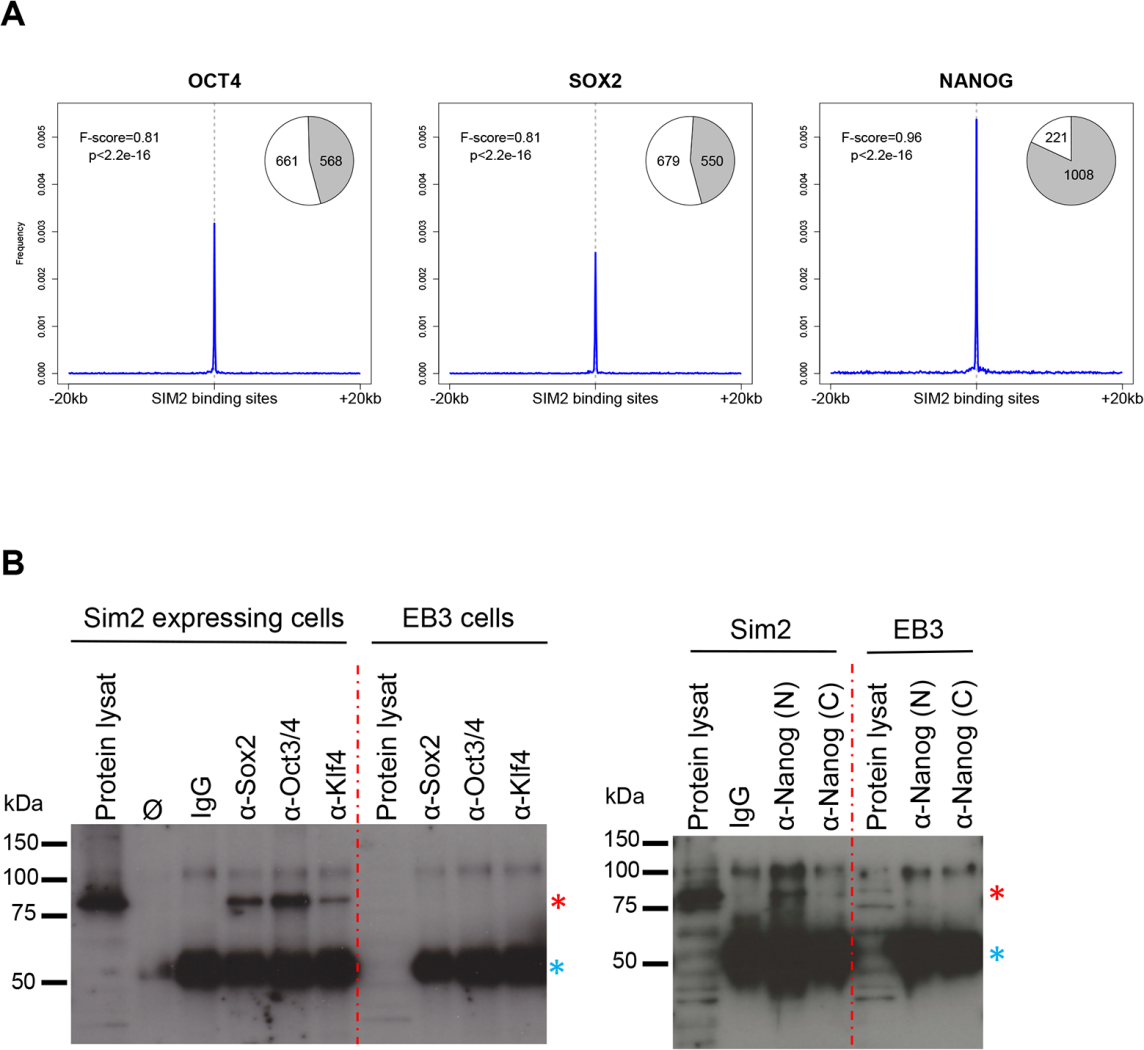
Overlapping SIM2 occupancy with master transcription factor binding sites. **a.** Frequency distribution of OCT4, SOX2 and NANOG DNA binding sites in a 40kb window centered to the newly identified SIM2 DNA binding sites. Plots show a significant enrichment for the OSN binding sites at the SIM2 peak localization in SIM2 A6 expressing cells. Pie charts show the proportion of SIM2 DNA binding sites overlapping with the OCT4, SOX2 or NANOG binding sites (in grey) (100bp window). p = Fisher’s exact test p-value; F score: measure of the significance of the association (1 = perfect match). **b.** Protein co-immunoprecipitation experiments of SIM2-FLAG with endogenous OCT4, SOX2, KLF4 (left panel) and NANOG (right panel). Cellular protein extracts from *Sim2* expressing cells (A6) or EB3 cells were immunoprecipitated by using antibodies directed against each of the pluripotency factors (N-terminal and C-terminal part of NANOG) or IgG as a negative control for co-immunoprecipitation. Associated proteins were immunoblotted using an anti-FLAG antibody. Red star shows the SIM2-FLAG protein, blue star the signal given by the recognition of the IgG heavy chains. Ø: Beads only; kDa: kilodaltons; protein lysat: protein lysat was loaded as an input control for the immunoblot.

The binding motifs similarities as well as the key role of these master transcription factors led us to further investigate the possible overlap between the regions occupied by SIM2 and the binding sites of the OSN factors in the ES cells. To do so, we first generated additional ChIP-sequencing data for OCT4, SOX2 and NANOG in the *Sim2* overexpressing cells (A6 clone) and the EB3 parental line. The numbers of binding sites identified for each factor are summarized in S6 Table. We investigated the distribution of those binding sites in a 40kb window around the SIM2 peaks. Interestingly, for the three factors, we found an increased frequency of binding sites at the localization of the SIM2 peaks, suggesting that SIM2 occupies preferentially the same genomic loci as these master transcription factors in the genome (Fig 5A). In the *Sim2* expressing cells, 82% of SIM2 peaks overlap with a DNA binding site for NANOG, 46.2% with a binding site for OCT4 and 44.75% with a binding site for SOX2 (100bp window) (Fig 5A). Comparison with a random set of peaks revealed that this overlap is significantly higher than expected by chance (p<2.2e-16). We validated those results by examining ChIP-Seq data previously published for OCT4, SOX2 and NANOG as well as other pluripotency factors including KLF4 and ESRRB [33]. This comparison revealed the same enrichment for the binding of these factors at the SIM2 peaks (S1 Fig). Altogether, these data suggest that SIM2 could co-occupy a number of loci with master transcription factors involved in the control of the pluripotent state. We performed protein co-immunoprecipitation experiments to test if SIM2 interacts with partners of the OSN protein complex. Importantly, detectable amounts of SIM2-FLAG were co-immunoprecipitated with antibodies against endogenous SOX2, OCT3/4, KLF4 and NANOG in a total cellular protein extract from *Sim2* expressing mES cells (Fig 5B and S2 Fig). These results support our hypothesis and suggest that SIM2 interacts independently with these four transcription factors. SIM2 might therefore be a partner of the OSN complex carrying a key regulatory role in ES cells.

### SIM2 marks enhancer and super-enhancer regions

**Fig 6:**
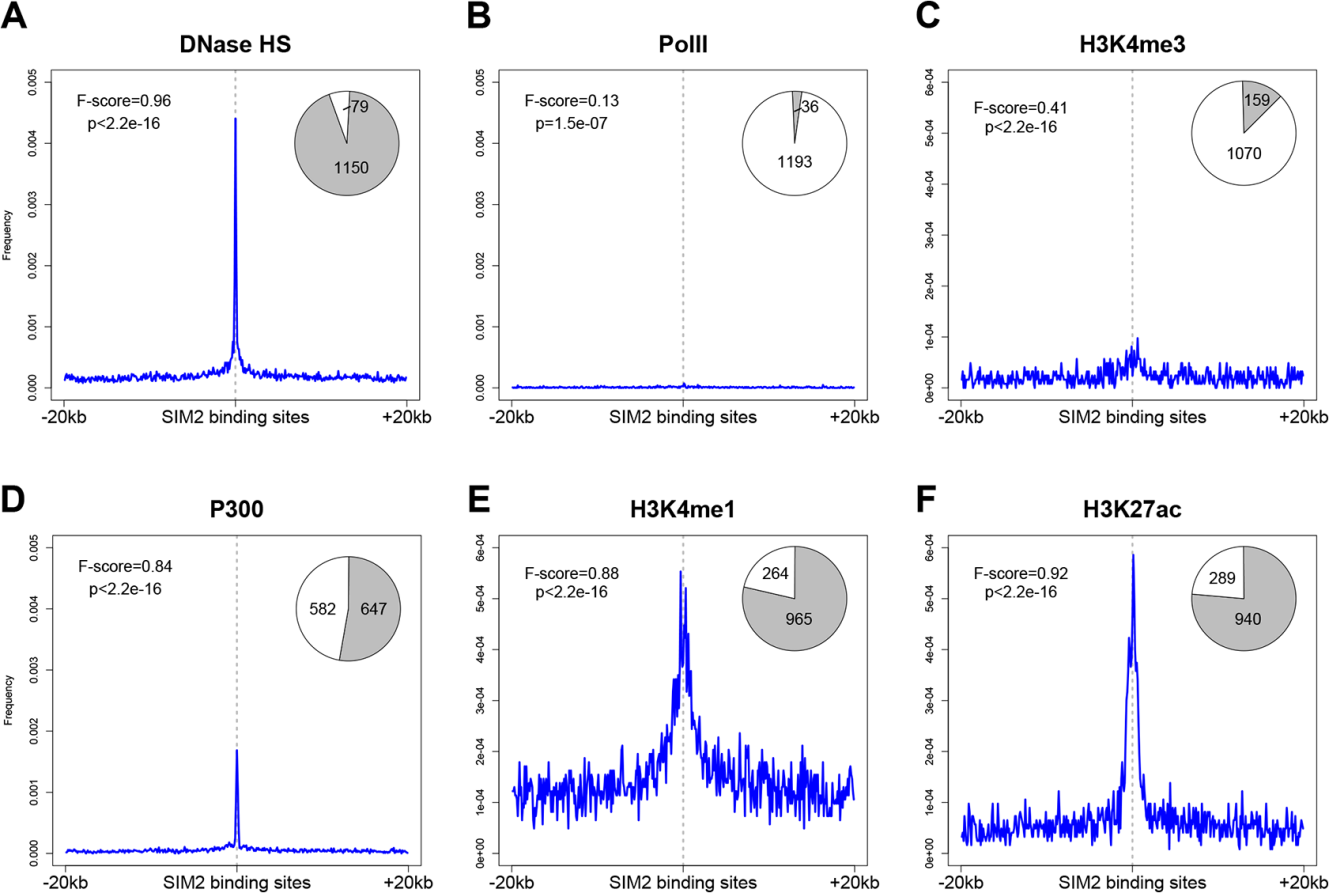
SIM2 DNA binding sites colocalize with known enhancer marks. Distribution of chromatin modification marks in a 40kb window centered to the SIM2 DNA binding sites: DNaseI hypersensitivity signal (**a**), RNA polymerase II (**b**), H3K4me3 (**c**), P300 (**d**), H3K4me1 (**e**) and H3K27ac (**f**). Pie charts show the proportion of SIM2 peaks overlapping each of these marks (in grey) (100bp window). p = Fisher’s exact test p-value; F score: measure of the significance of the association (1 = perfect match). Data were taken from the mouse ENCODE project in the UCSC genome browser mm9 build (http://genome.ucsc.edu/).

A previous study has described the co-binding of the OSN master transcription factors in ES cells as predictive for enhancer activity [33]. In order to test if the binding of SIM2 could also predict such cis-regulatory activity, we analyzed the distribution of chromatin modification marks (available from the mouse ENCODE project) in the vicinity of the SIM2 binding sites. We first observed a significant increase of chromatin accessibility at the loci occupied by SIM2, as revealed by the enrichment for DNaseI hypersensitivity (HS) (Fig 6A). We then examined the distribution of P300, H3K4me1 (monomethylation of lysine 4 of histone 3) and H3K27ac (acetylation of lysine 27) to investigate the enhancer profile in the genomic regions bound by SIM2. We found that the SIM2 binding regions significantly overlap with these enhancer marks, suggesting that the presence of SIM2 may coincide with an enhancer activity, as previously described for the pluripotency factors (Fig 6D–6F). Consistently, SIM2 binding sites were found to significantly overlap with the typical enhancers described by Whyte *et al*. [44] (S1 Fig). In contrast, marks for promoter signals such as RNA polymerase II occupancy or H3K4me3 (trimethylation of lysine 4) were poorly enriched, suggesting that SIM2 cannot extensively predict a promoter activity (Fig 6B and 6C).

**Fig 7:**
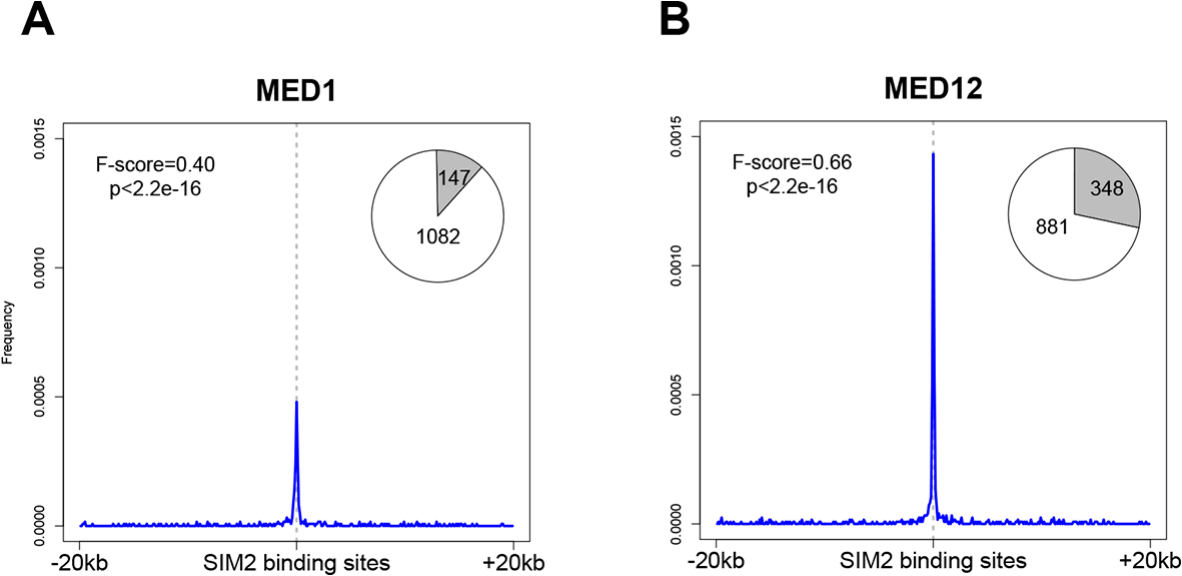
The Mediator complex colocalizes with the SIM2 DNA binding sites. Frequency distribution of MED1 (**a**) and MED12 (**b**) DNA binding sites in a 40kb window centered to the SIM2 peaks. Pie charts show the proportion of SIM2 DNA binding sites overlapping MED1 or MED12 DNA binding sites (in grey) (100bp window). p = Fisher’s exact test p-value; F score: measure of the significance of the association (1 = perfect match).

A recent study reported the existence of a sub-category of enhancers known as super-enhancers [44]. We found that a significant fraction of Sim2 binding sites overlap with those (S1 Fig) and thus further investigated this correlation. These super-enhancer regions are characterized by the co-occupancy of OCT4, SOX2 and NANOG. They mainly differ from the typical enhancers by the length of the DNA regions they span and by the increased presence of the Mediator coactivator complex. Additionally, they possess a specific transcription factor signature enriched for KLF4 and ESRRB but excluding other ES cell factors such as CTCF or c-Myc. Interestingly, by examining the binding profile of all these factors (data taken from Chen *et al*. [33]) in the genomic regions occupied by SIM2, we found indeed that CTCF and c-Myc were not enriched as opposed to KLF4 and ESRRB (S1 Fig). We performed ChIP-sequencing to investigate the binding genomic regions of MED1 and MED12, the main constituents of the Mediator complex, in the *Sim2* expressing cells. We revealed a significant overlap between SIM2 binding sites and each of these factors (p-value<2.2E-16, Fig 7). ChIP-seq data available for the MED1 protein [34] confirmed the significant enrichment at the SIM2 binding sites (S1 Fig). Altogether, these results suggest that SIM2 is implicated in conventional enhancers as well as in regulatory functions of super-enhancers.

## Discussion

In this study, we have shown how the identification and characterization of the SIM2 DNA binding sites improve the understanding of its molecular function and potential role in the manifestations of DS.

### SIM2 target genes confirmed the contribution of SIM2 to the DS cognitive impairment

We have identified 1229 binding loci for SIM2 and shown that a significant fraction of target genes located in the vicinity were involved in neuronal development processes. These results suggest that SIM2 may be a candidate gene for some DS phenotypes, in particular the cognitive impairment. These findings validate the hypotheses established so far with studies performed in Drosophila and in mouse models [6–8, 19–23]. In addition, our study enabled the discovery of target genes involved in mechanisms of transcription regulation revealing that SIM2 may have a role of master transcription factor and act upstream of important mechanisms controlling the gene expression in mES cells. By combining RNAPII ChIA-PET data, SIM2 binding sites and the transcriptome analysis in *Sim2* expressing cells, we have established a set of 22 genes that could be considered as direct targets of SIM2. Among them, several genes are known to be involved in molecular functions that could possibly be related to DS manifestations. For instance, the *OTX2* gene (Orthodenticle Homeobox 2) is known as an important transcription factor for the control of brain and craniofacial development [45]. Mutations of this gene have been linked to craniofacial malformations in both mouse and human [46, 47]. Patients harboring *OTX2* mutations present a microphthalmia syndrome associated to multiple features resembling those of DS such as developmental delay, hypotonia or short stature [48]. Similarly, the *ARID1B* gene (AT Rich Interactive Domain 1B), a member of the SWI/SNF chromatin remodeling complex, has been recently associated to cognitive impairment [49] and more specifically to the Coffin-Siris syndrome characterized by intellectual disability, severe speech delay and typical facial features [50, 51]. Finally, the *SYNGR1* gene (Synaptogyrin 1) also constitutes an interesting target gene given its role in synaptic plasticity, as revealed by the *Syngr1* knockout mice [52].

### Overexpression of SIM2 may influence the pluripotency signature of mES cells

The enrichment for enhancer marks at the SIM2 binding loci as well as the relatively small number of peaks located in promoter regions show that SIM2 is mainly recruited to distant regulatory elements for the regulation of its target genes. Here, we reported that SIM2 could bind genomic loci occupied by master transcription factors involved in the control of ES cell pluripotency. Co-immunoprecipitation experiments even suggest that SIM2 can physically interact with these factors and raise the possibility that they are part of the same protein complex. Interestingly, a study using the Drosophila model validated this hypothesis by showing functional interactions between SIM, SOX and POU transcription factors for the control of midline gene expression [53]. An interesting hypothesis has recently been developed regarding the role of pioneer transcription factors in the cells [54]. Indeed, it is well assumed that the recruitment of transcription factors is highly dependent on the chromatin state and that epigenetic modifications will likely influence their binding on the target sequences. Pioneer factors are known to act upstream of classical transcription factors to promote their binding on enhancer regions by modifying the chromatin landscape in order to improve its accessibility. The Forkhead box protein A1 (FOXA1) constitutes a typical example of pioneer factor acting during neuronal differentiation by changing enhancer chromatin signatures to promote the binding of subsequent factors [55, 56]. Interestingly, it has been proposed that OCT4, SOX2 and KLF4 could play a role of pioneer factors at distal enhancers during pluripotency reprogramming [57–59]. Thus, we can hypothesize that the enhancer sequences bound by SIM2 may initially be occupied by pioneer factors such as OCT4, SOX2, NANOG or KLF4 to modify the chromatin structure and facilitate the recruitment of SIM2 in response to specific differentiation signals.

This colocalization also raises the possibility that SIM2 interferes with the binding of the master transcription factors and thus could modify the pluripotent state of mES cells. This function has been previously reported for other factors including CDX2 (Caudal type homeobox 2) [60]. Indeed, it was shown that CDX2 has the ability to interfere with the binding of OCT4, SOX2 and NANOG, inducing the downregulation of their target genes. Since these pluripotency factors are known to control their own expression through auto-regulatory loops, it is likely that the binding interference of CDX2 contributes to the OSN downregulation and thus to the initiation of differentiation processes. The same type of mechanisms could be proposed to explain the function of SIM2, especially since the *Sox2*, *Nanog*, *Klf4* and *Esrrb* genes belong to the list of targets associated to SIM2 binding sites in our results.

Interestingly, we have observed that SIM2 can also mark a particular subtype of enhancers called super-enhancers. Those are known to be associated with genes, mostly transcription factors, essential for the maintenance of the ES cell identity [44]. This observation raises the hypothesis that the binding of SIM2 in super-enhancer regions could modify the sensitive balance controlling the transcription program of ES cells and then promote the transition towards specific pathways, most probably neuronal differentiation. Consistently, several transcriptome studies have shown an increased expression of SIM2 in the early stages of the neuronal differentiation [61–63]. The mechanisms responsible for this transition are probably tightly controlled and we hypothesize that the dysregulation of SIM2 could disturb this fragile equilibrium. Further experiments will certainly help to understand the role of SIM2 in the differentiation processes.

Our data open interesting perspectives for the understanding of the mechanisms underlying the DS phenotypes and emphasize the benefit of using an ES cell model to study the function of HSA21 transcription factors.

## Supporting Information

S1 Fig Frequency distribution of published transcription factor binding sites, typical enhancers and super-enhancers in a 40kb window around the SIM2 peaks. Pie charts give the number of SIM2 peaks overlapping with the binding sites of each of the transcription factors, typical enhancers or super-enhancers (in grey) (100bp window). Typical enhancers and super enhancers data were taken from Whyte *et al*. [44]. MED1 ChIP-seq data were taken from Kagey *et al*. [34] and other data from Chen *et al*. [33].
(TIF)

S2 Fig Protein co-immunoprecipitation experiments of SIM2-FLAG with endogenous OCT4, SOX2, KLF4 (left panel) and NANOG (right panel) (replication of the experiment shown Fig 4B). Cellular protein extracts from *Sim2* expressing cells (A6) or EB3 cells were immunoprecipitated by using antibodies directed against each of the pluripotency factors (N-terminal and C-terminal part of NANOG) or IgG as a negative control for co-immunoprecipitation. Associated proteins were immunoblotted using an anti-FLAG antibody. Red star shows the SIM2-FLAG protein, blue star the signal given by the recognition of the IgG heavy chains. Ø: Beads only; kDa: kilodaltons; protein lysat: protein lysat was loaded as an input control for the immunoblot.
(TIF)

S1 Table List of Sim2 DNA binding sites. (XLSX)

S2 Table Gene Ontology analysis on the putative Sim2 targets. (XLSX)

S3 Table List of differentially expressed genes. (XLSX)

S4 Table List of Sim2 target genes dysregulated in *Sim2* expressing cells. (XLSX)

S5 Table ChIA-PET interactions. (XLSX)

S6 Table Number of Oct4, Sox2 and Nanog binding sites in the Sim2 expressing cells and the EB3 parental line. (PDF)
