# Supplementary figures and images for: HSA21 Single-Minded 2 (Sim2) Binding Sites Co-Localize with Super-Enhancers and Pioneer Transcription Factors in Pluripotent Mouse ES Cells
Source: PLoS One. 2015 May 8;10(5):e0126475. doi: 10.1371/journal.pone.0126475 (PMC4425456; doi:10.1371/journal.pone.0126475)

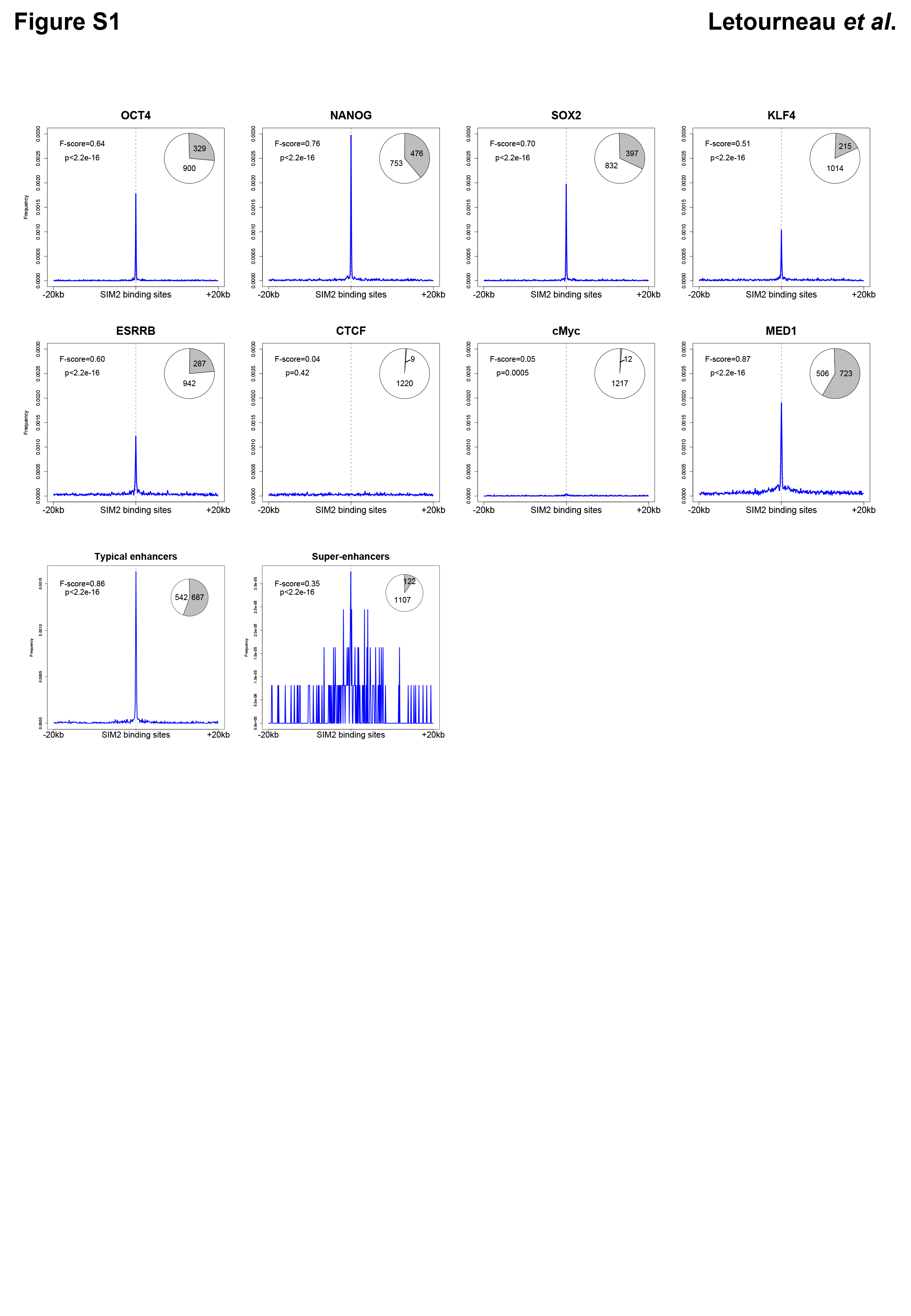

Supplement: S1 Fig — Pie charts give the number of SIM2 peaks overlapping with the binding sites of each of the transcription factors, typical enhancers or super-enhancers (in grey) (100bp window). Typical enhancers and super enhancers data were taken from Whyte et al. [44]. MED1 ChIP-seq data were taken from Kagey et al. [34] and other data from Chen et al. [33]. (TIF) [file pone.0126475.s001.tif]

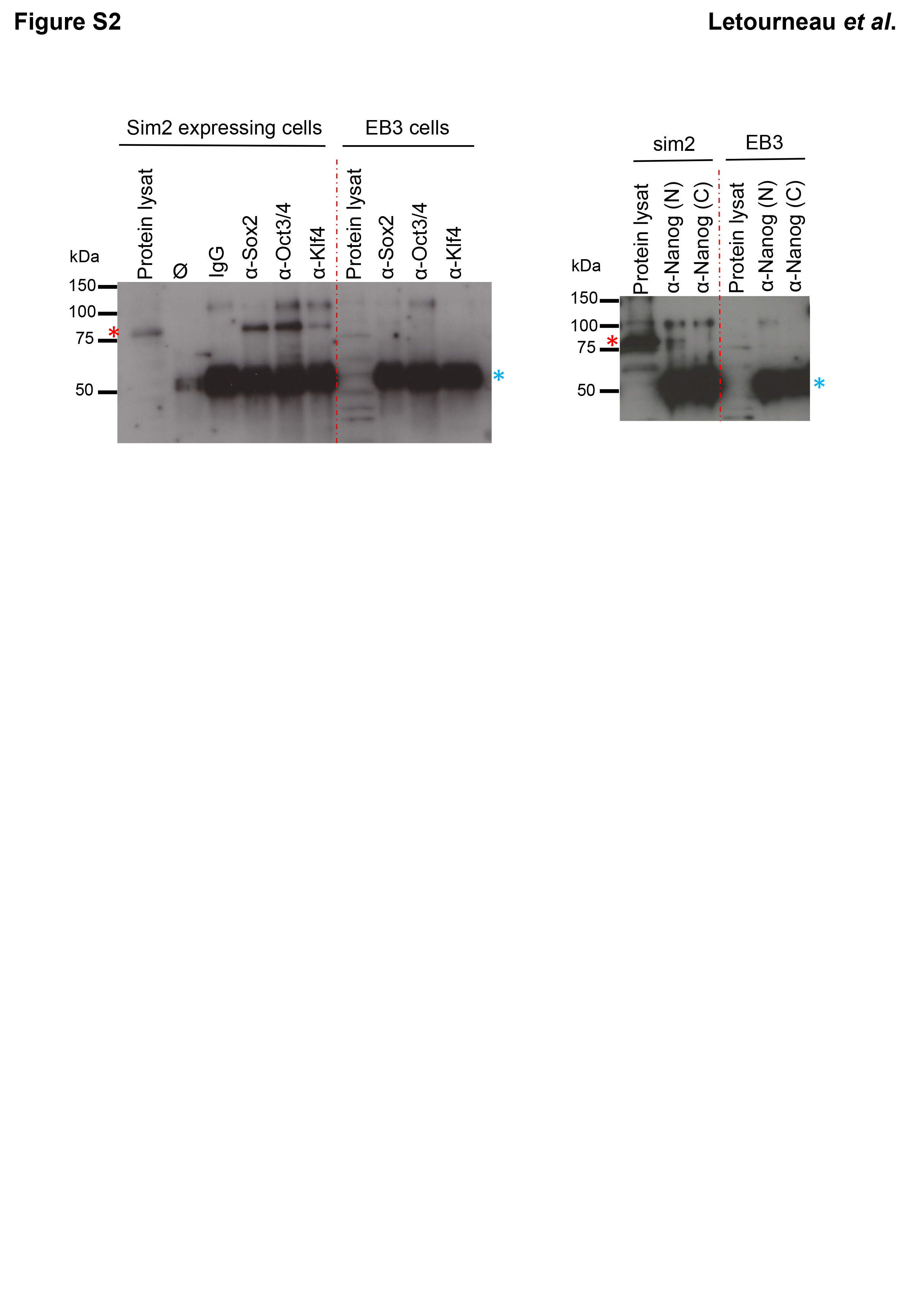

Supplement: S2 Fig — Cellular protein extracts from Sim2 expressing cells (A6) or EB3 cells were immunoprecipitated by using antibodies directed against each of the pluripotency factors (N-terminal and C-terminal part of NANOG) or IgG as a negative control for co-immunoprecipitation. Associated proteins were immunoblotted using an anti-FLAG antibody. Red star shows the SIM2-FLAG protein, blue star the signal given by the recognition of the IgG heavy chains. Ø: Beads only; kDa: kilodaltons; protein lysat: protein lysat was loaded as an input control for the immunoblot. (TIF) [file pone.0126475.s002.tif]
