# Supplementary material for: HSA21 Single-Minded 2 (Sim2) Binding Sites Co-Localize with Super-Enhancers and Pioneer Transcription Factors in Pluripotent Mouse ES Cells
Source: PLoS One. 2015 May 8;10(5):e0126475. doi: 10.1371/journal.pone.0126475 (PMC4425456; doi:10.1371/journal.pone.0126475)
Supplement: S6 Table — (PDF) [file pone.0126475.s008.pdf]

	OCT4	SOX2	NANOG
***Sim2*-expressing cells**	2’532	2’196	14’815
**EB3 parental line**	3’880	3’614	11’368
